# PathNet: a tool for pathway analysis using topological information

**DOI:** 10.1186/1751-0473-7-10

**Published:** 2012-09-24

**Authors:** Bhaskar Dutta, Anders Wallqvist, Jaques Reifman

**Affiliations:** 1 DoD Biotechnology High Performance Computing Software Applications Institute, Telemedicine and Advanced Technology Research Center, U.S. Army Medical Research and Materiel Command, Ft. Detrick, MD, 21702, USA

**Keywords:** Canonical pathways, Pathway enrichment, Pathway association, Pathway interaction, Pathway topology

## Abstract

**Background:**

Identification of canonical pathways through enrichment of differentially expressed genes in a given pathway is a widely used method for interpreting gene lists generated from high-throughput experimental studies. However, most algorithms treat pathways as sets of genes, disregarding any inter- and intra-pathway connectivity information, and do not provide insights beyond identifying lists of pathways.

**Results:**

We developed an algorithm (PathNet) that utilizes the connectivity information in canonical pathway descriptions to help identify study-relevant pathways and characterize non-obvious dependencies and connections among pathways using gene expression data. PathNet considers both the differential expression of genes and their pathway neighbors to strengthen the evidence that a pathway is implicated in the biological conditions characterizing the experiment. As an adjunct to this analysis, PathNet uses the connectivity of the differentially expressed genes among all pathways to score pathway contextual associations and statistically identify biological relations among pathways. In this study, we used PathNet to identify biologically relevant results in two Alzheimer’s disease microarray datasets, and compared its performance with existing methods. Importantly, PathNet identified de-regulation of the ubiquitin-mediated proteolysis pathway as an important component in Alzheimer’s disease progression, despite the absence of this pathway in the standard enrichment analyses.

**Conclusions:**

PathNet is a novel method for identifying enrichment and association between canonical pathways in the context of gene expression data. It takes into account topological information present in pathways to reveal biological information. PathNet is available as an R workspace image from http://www.bhsai.org/downloads/pathnet/.

## Background

High-throughput technologies enable the study of biological processes at the systems level. However, analyzing the large amount of data generated by high-throughput techniques and translating these data into biological knowledge is currently a critical bottleneck in systems biology. To study a disease at the system level, DNA microarrays are routinely used to provide a comparison of gene expression patterns in control vs. disease conditions. Because this comparison usually reveals a large number of differentially expressed genes, it is difficult, if not impossible, to analyze the effect of each gene individually. In addition, high-throughput data often contain considerable noise, making individual or isolated gene observations less likely to be relevant. Using statistical methods to summarize the data can help reduce noise and increase the reproducibility of the results [1]. However, translating these results into biological knowledge remains challenging.

The most commonly used methods for summarizing gene expression data rely on enrichment analysis of differentially expressed genes to identify and rank Gene Ontology (GO) terms and canonical pathways in order to characterize the underlying biological nature of the data. Comprehensive reviews of these approaches are available [2-4]. While the hierarchically ordered GO terms describe the properties of gene products, canonical pathways describe the connectivity between genes and gene products involved in a given biological process. The simplest and most widely used method for identifying pathways based on gene expression data is the hypergeometric test [5], which assesses whether the number of differentially expressed genes in a pathway is significantly higher than what would be expected by chance. A popular alternative to the hypergeometric test for assessing the relevance of pathways is the gene set enrichment analysis (GSEA) [6]. This method considers the relative positions of pre-defined gene sets (pathways) in a rank-ordered list of differentially expressed genes, in order to determine if a pathway is relevant to the experimental study.

Well-studied canonical pathways provide extensive information about how the genes and gene products interact and regulate each other. However, most of the pathway analysis methods, including the hypergeometric test and GSEA, treat pathways as lists of genes and do not take into account the connectivity information embedded within the pathway. More recently, some studies [7-9] have included such topological information for calculating enrichment of signaling pathways, by assigning different weights to genes based on their location in the pathway. Nevertheless, these methods still consider each pathway as an isolated entity, where, in reality, pathways are not isolated; they may share genes. In fact, out of 130 non-metabolic pathways from the Kyoto Encyclopedia of Genes and Genomes (KEGG) database [10], 88 pathways have 20% or fewer genes unique to that pathway, while only 6 pathways have 80% or more unique genes. In fact, all pathways shared at least one gene with another pathway. Thus, to fully take into account the biological information collected and encoded in a database such as KEGG, all pathways should be pooled together to allow for exploitation of inter-pathway connectivity information. However, none of the current methods for pathway analysis incorporates intra- and inter-pathway connectivity information for enrichment analysis.

In this study, we have attempted to address these issues by developing an algorithm for examining pathway enrichment that uses differential gene expression (or other molecular profiling data) to analyze Pathways based on Network information (PathNet). To incorporate inter-pathway connectivity, we combined KEGG pathways (from http://www.kegg.com) to create a *pooled pathway*. For enrichment analysis, PathNet first identifies the association of each gene with a disease (referred to as direct evidence) by comparing gene expression data in control patients vs. patients with the disease. Then, PathNet identifies the association of each gene’s neighbors with the disease (referred to as indirect evidence) based on the inter- and intra-pathway connectivity information present in the *pooled pathway*. Finally, PathNet combines the direct and indirect evidences to obtain the significance of the combined evidence. Based on the statistical significance of the combined evidence for all genes, PathNet uses the hypergeometric test to uncover the pathways associated with the disease.

As genes in pathways function in a coordinated fashion, association studies between pathways in the context of gene expression data can unravel the underlying complexity of biological processes. Li *et al.*[11] proposed that pathways are more likely to interact when the number of protein-protein interactions (PPI) between proteins from two pathways are greater than what would be expected by chance. Based on this assumption, they create a network of pathways and identify the activated pathway modules in a given study by mapping the gene expression data enriched pathways onto the network. Recently, Kelder *et al.*[12] identified indirect associations between pathways by integrating pathway information, PPI networks, and gene expression data. Liu *et al*. [13] estimated crosstalk by mapping gene expression on PPIs between proteins from the Alzheimer’s disease (AD) pathway and other pathways sharing genes with the AD pathway. As PPI networks are usually noisy, identifying indirect associations using PPI network might produce false positive associations. In contrast with other approaches, PathNet assesses the association in the context of gene expression data based on intra- and inter-pathway connectivity in the *pooled pathway*. This association of specific pathways, beyond the mere overlap of genes annotated as belonging to more than one pathway, can reveal otherwise hidden pathway dependencies (and hence biological insights) that are not directly attainable from enrichment analysis alone.

To illustrate the utility of PathNet, we applied it to two AD microarray datasets and analyzed the results in the context of existing knowledge. In addition, we show how the statistical scores of the associations between pathways through gene expression data facilitated the identification of a biological association between the AD pathway and ubiquitin-meditated proteolysis pathway.

## Methods

### Pathway network from KEGG pathways

Pathways from the KEGG database [10] available in November 2010 were downloaded as KEGG Markup Language files. Each of the 130 non-metabolic pathways present in the KEGG database were represented as directed graphs, where the nodes and edges of a graph were, respectively, characterized by unique gene IDs and interactions in the pathway. KEGG interactions representing processes, such as phosphorylation, dephosphorylation, activation, inhibition, and repression, were accounted for by directed edges, whereas bidirectional edges were used to represent binding/association events. The complete mapping between edge directionality and KEGG protein interaction attributes is provided in Additional file 1. All 130 pathways were combined to create a *pooled pathway*, and the R package, named ‘An interface to the BOOST graph library,' from Bioconductor (http://www.bioconductor.org/packages/rel-ease/bioc/html/RBGL.html) was used to convert this information into the adjacency matrix (A). The adjacency matrix is a non-symmetric square matrix, where the number of rows (and columns) represents the number of genes present in the *pooled pathway*. The diagonal elements of matrix A were set to zero to exclude self-interactions. The non-diagonal element A_ij_ represents the directed KEGG protein interaction between nodes *i* and *j:*

(1) $$\;A_{\mathrm{ij}}=\{\begin{array}{c} 1\;\text{if there is an interaction from node}\;i\;\text{to node}\;j\;\\ 0\;\text{otherwise}\; \end{array}$$

In the case of a bidirectional interaction, two edges are introduced, one from node *i* to node *j* and another from node *j* to node *i*. Although the bulk of the genes annotated in KEGG pathways are present on most microarray chips, about 10% of the genes are typically missing. In order to only include information derived from experimental data, we re-constructed the adjacency matrix for each chip-set by deleting rows and columns of genes that were not examined experimentally. In order to be consistent in the analysis presented below, we also redefined the *pooled pathway* for each chip-set to include only genes for which experimental data exists. PathNet automatically carries out this step from the input files.

### Pathway enrichment analysis

**Figure 1:**
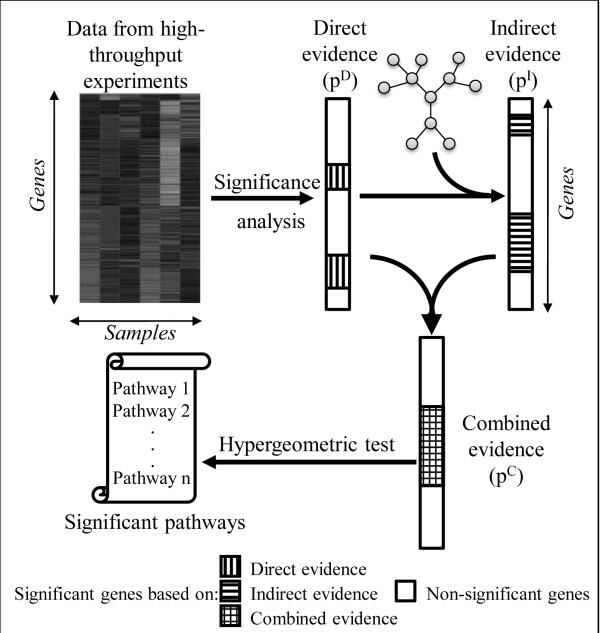
**Schematic representation of PathNet analysis.** The direct evidence pertaining to differential gene expression is detected via microarray analysis while the indirect evidence of a gene is calculated from the direct evidences of its neighbors in the pathway. The direct and indirect evidences are combined, and the combined evidence is used to identify pathway enrichments via a hypergeometric test. The combined input of microarray data and pathway information yields a final pathway enrichment list that can be associated with the different test conditions in the samples.

PathNet combines two types of evidence for pathway enrichment analysis, referred to as direct evidence and indirect evidence (Figure 1). Direct evidence accounts for the differential expression of gene *i* between two experimental conditions (control and disease), while indirect evidence considers the differential expression of the neighbors of gene *i* in the *pooled pathway*. The nominal p-values associated with the direct and indirect evidences of each gene were combined to obtain the p-value of the combined evidence*,* which is subsequently used for the pathway enrichment analysis.

We used the t-test to calculate a nominal p-value for the direct evidence (p_i_^D^) in order to gauge whether the average expression of gene *i* was different between the two experimental conditions. The lower the p^D^-value, the more likely it is that the observed difference in gene expression is significant. Alternative methods, such as SAM [14] or ANOVA [15], can also be used to estimate p^D^.

To ascertain the significance of the indirect evidence, we need to test whether the expression of each neighbor of gene *i* is or is not different between the two experimental conditions. To characterize this difference, we first calculated the indirect evidence score (SI_*i*_), which incorporates the topological information of the pathways. This score captures a weighted level of differential expression of the neighbors of gene *i,* and is calculated using the following equation:

(2) $$SI_{i}=\underset{j\in G,i\neq j}{\sum }A_{\mathrm{ij}}*\left(-log_{10}\left(p_{j}^{D}\right)\right)\;$$

where G denotes the set of all genes present in the *pooled pathway*, A_ij_ is defined as in Eq. (1), and p_j_^D^ denotes the nominal p-value of the direct evidence for gene *j* which is used to assign the weight of the contribution. The nominal p-value associated with the indirect evidence (p_i_^I^) was inferred by testing if the observed score SI_i_ was greater than the corresponding random values created by shuffling the p_j_^D^-values in the *pooled pathway*. In each of the N shuffles, all p_j_^D^-values were scrambled by randomly re-assigning their indices. As the connectivity in the *pooled pathway* remained fixed, for each gene *i* in the n^th^ shuffle, we calculated the corresponding random score SI_i_^R^(n). Next, for each gene *i*, we formally re-constructed the probability density distribution function for the random scores p_i_^R^. Practically, we estimated the p_i_^I^-values by counting the number of random scores larger than the actual scores, as follows:

(3) $$p_{i}^{I}\equiv \overset{\infty }{\underset{SI_{i}}{\int }}P_{i}^{R}\left(x\right)dx\approx \frac{1}{N}\overset{N}{\underset{n=1}{\sum }}\{\begin{array}{c} 1\;\text{if}\;SI_{i}^{R}\left(n\right)>SI_{i}\\ 0\;\text{otherwise}\; \end{array}\;$$

In our calculations, we used N = 2,000 shuffles. As the estimated p_i_^I^-values are integer multiples of 1/N, we cannot accurately estimate p_i_^I^-values if they are less than 1/N. To address this issue, we assigned 1/N as the minimum p_i_^I^-value. The lower the p_i_^I^-value, the more likely it is that the observed weighted gene expression pattern around gene *i* is not a random pattern.

We obtained the p-value of the combined evidence (p_i_^C^) for each gene *i* by using Fisher’s method [16] to aggregate the nominal p-values associated with the direct and indirect evidences (p_i_^D^ and p_i_^I^). Previous studies [17,18] have shown that this method is optimal for combining independent p-values, when compared to other methods. In our case, the indirect evidence associated with a gene is dependent only on the magnitude of the differential gene expression of its neighbors, and not on its own expression levels, which formally ensures independence between the p-values. Additional file 2 shows p^D^- versus p^I^-values for the datasets we used and there was no obvious dependency of these values on each other. We also verified that the set of p^D^- and p^I^-values were linearly independent for all comparisons by calculating a non-significant correlation coefficient in each test set. Accordingly, for gene *i*, the two probabilities were combined based on Fisher’s method, using the following equation:

(4) $$p_{i}^{C}=\overset{\infty }{\underset{-2ln\left(p_{i}^{D}*p_{i}^{I}\right)}{\int }}P\left(\chi_{4}^{2}\right)$$

where P(χ_4_^2^) denotes the probability density function of the χ^2^ distribution with 4 degrees of freedom. Note that, even if the p^D^- and p^I^-values were correlated, they could still be combined using a modified version of Fisher’s method [19].

For genes that are isolated and not connected in any pathway, there are no p^I^-values to consider, hence p^C^ = p^D^. Finally, we selected genes with p_i_^C^ < 0.05 as differentially expressed and used the hypergeometric test to calculate pathway enrichment. For all hypergeometric tests, we used the ‘phyper’ function of the R programming language.

### Contextual association between pathways

**Figure 2:**
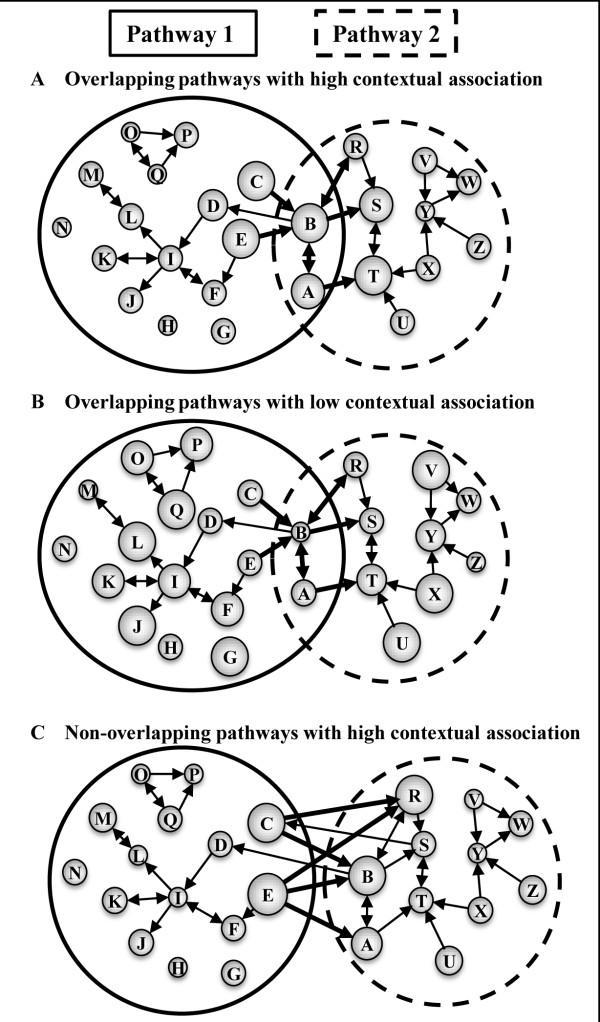
**Schematic representation of three scenarios of contextual association between pathways.** Each illustration (**A-C**) shows two pathways (sets of both connected and isolated genes inside ellipsoids) with varying degrees of overlapping genes. The size of the circles (genes) represents the level of differential gene expression between control and disease patients (the higher the significance associated with the expression change, the larger the circle). The lines and arrows represent KEGG-derived interactions between genes as annotated in the *pooled pathway*, and the thick lines represent edges connecting genes from Pathway 1 to Pathway 2. (**A**) Overlapping pathways with high contextual association. Whereas Pathway 1 and Pathway 2 can be associated because genes *A* and *B* are shared between the two pathways, the fact that overlapping genes are directly connected to other differentially expressed genes (thick connections to large circles) enhances this association. (**B**) Overlapping pathways with low contextual association. The pathway maps are exactly the same as in case (**A**). However, Pathway 1 is “less” associated with Pathway 2 in the context of gene expression data, as the genes connected by thick edges are modestly differentially expressed (thick connections to small circles). (**C**) Non-overlapping pathways with high contextual association. Although, Pathway 1 and Pathway 2 do not share any genes, genes from these two pathways are connected based on inter-pathway connectivity inferred from the *pooled pathway.* Pathway 1 is contextually associated with Pathway 2 because the genes connecting these two pathways are differentially expressed.

As discussed above, KEGG pathways are not isolated; some genes are shared between pathways. Thus, differential gene expression in one pathway may be directly linked to differential gene expression in another pathway. Whereas the existing pathway annotations provide a static association among genes and pathways, gene expression data for particular conditions provide context-dependent information. Here, we considered all connections in the *pooled pathway* to identify possible contextual pathway-pathway associations based on a weighted measure of differential gene expression among shared pathway genes. Figure 2 outlines three ways in which differential gene expression data can link two pathways that either directly share genes or are linked via gene connections annotated in other pathways.

We calculated the contextual score SC_αβ_ to quantify the biological association via differentially expressed genes from the *pooled pathway*, between two pathways α and *β*. The SC_αβ_ from pathway α to pathway *β* is calculated using the following equation:

(5) $$\begin{array}{cc} \;SC_{\alpha \beta } & =\underset{i\in g^{\alpha }}{\sum }\underset{j\in g^{\beta }}{\sum }A_{\mathrm{ij}}*\left(-log_{10}\left(p_{i}^{D}\right)\right)\\ & \;*\left(-log_{10}\left(p_{j}^{D}\right)\right) \end{array}$$

where g^α^ and g^β^ denote the set of genes in pathway α and *β*, respectively, A_ij_ is defined as in Eq. (1), and p_i/j_^D^ denotes the nominal p-value of the direct evidence for gene *i/j* used to construct the weight for each A_ij_ value. Note that as A_ii_ *≡* 0, the SC_αβ_ does not contain self interactions and only includes gene pairs that have been connected to each other via the *pooled pathway*. The formulation uses only the p^D^-values associated with the direct evidence and not the p^C^-values, which already contain pathway information via the indirect evidence as calculated in Eq. (2). A higher SC_αβ_ indicates a stronger contextual association between the pathways.

To evaluate the probability of finding a SC_αβ_ greater than expected by chance alone, we followed the same procedure used to estimate the p-values for the indirect evidence. The p-value associated with the SC_αβ_ (p_αβ_) was inferred by testing if the observed score SC_αβ_ were greater than the corresponding random values created by shuffling all the p^D^-values in the *pooled pathway N* times. With the connectivity in the *pooled pathway* fixed, for each pathway pair α and *β* in the *n*^th^ shuffle, we calculated the corresponding random score SC_αβ_^R^(n). We then formally re-constructed, for each pathway pair α and *β,* the probability density distribution function for the random scores P_αβ_^R^. Finally, we estimated the p_αβ_-values by counting the number of random scores larger than the actual scores for each pathway pair:

(6) $$p_{\alpha \beta }\equiv \overset{\infty }{\underset{SC_{\alpha \beta }}{\int }}P_{\alpha \beta }^{R}\left(x\right)dx\approx \frac{1}{N}\overset{N}{\underset{n=1}{\sum }}\{\begin{array}{c} 1\;\text{if}\;SC_{\alpha \beta }^{R}\left(n\right)>SC_{\alpha \beta }\\ 0\;\text{otherwise}\; \end{array}$$

We used *N* = 2,000 shuffles to estimate the p_αβ_-values. The lower the p_αβ_-value, the more likely it is that the observed weighted gene expression pattern connecting pathways α and *β* are not a random pattern.

We also tested the extent to which the genes from pathways α and *β* overlap, based on common genes between the pathways. This information is only based on the KEGG database and is not dependent on gene expression data, i.e.*,* we used the full complement of KEGG genes to estimate this overlap. The hypergeometric test was used to estimate if the observed overlap was statistically significant.

### Microarray datasets

We evaluated the performance of the PathNet algorithm using two microarray datasets generated by two different research groups. Both datasets were downloaded from the National Center for Biotechnology Information’s Gene Expression Omnibus (GEO) database (http://www.ncbi.nlm.nih.gov/geo/) and involved AD-related studies. The first dataset (GEO ID: GDS810) [20], which we refer to as the *disease progression dataset,* investigated the expression profile of genes from the hippocampal region of the brain as a function of the progression of the disease (incipient, moderate, and severe). We refer to the second dataset [21] as the *brain regions dataset.* This dataset examined the effect of AD in six different brain regions: the entorhinal cortex, hippocampal field CA1, middle temporal gyrus, posterior cingulate cortex, superior frontal gyrus, and primary visual cortex (GEO ID: GSE5281). Because different regions of the brain are involved in controlling different biological processes, this dataset can provide insights into the tissue-specific activation of pathways. The entorhinal cortex region samples were obtained from patients in the early stages of AD, while the remaining samples were obtained from patients in the later stages of the disease.

In the *disease progression dataset*, the expression of each gene in patients with incipient, moderate, and severe disease was compared with control patients using the t-test. In the *brain regions dataset*, gene expression was compared between diseased and control patients for each brain region. We applied the proposed pathway enrichment method for each of these nine comparisons (three from the *disease progression dataset* and six from the *brain regions dataset*).

## Results and discussions

### Comparison of PathNet with existing algorithms in identifying pathways biologically relevant to AD

We used PathNet to identify the enrichment of pathways in each of the nine comparisons described above. We also compared the results of PathNet with three existing algorithms for pathway analysis that are currently in wide use: the hypergeometric test [5]; gene set enrichment analysis (GSEA) [6]; and signaling pathway impact analysis (SPIA) [8]. The GSEA and SPIA packages were downloaded from the Broad Institute (http://www.broadinstitute.org/gsea/index.jsp) and Bioconductor (http://www.bioconductor.org) Web sites, respectively. For GSEA, we used the provided Java-version of the program with a pre-ranked gene list. To ensure the comparability of results, we used the same version of the KEGG pathways (downloaded in November 2010) for all comparisons. Finally, to account for multiple comparisons, we corrected the pathway enrichment p-values for family-wise error rate (corrected p-values are represented as p_FWER_) and used a significance threshold of 0.05 for all comparisons. The results of all nine comparisons using each of the four pathway analysis methods are provided in Additional file 3, Additional file 4, and Additional file 5. Here, we summarize the results and the biological relevance of our findings.

**Figure 3:**
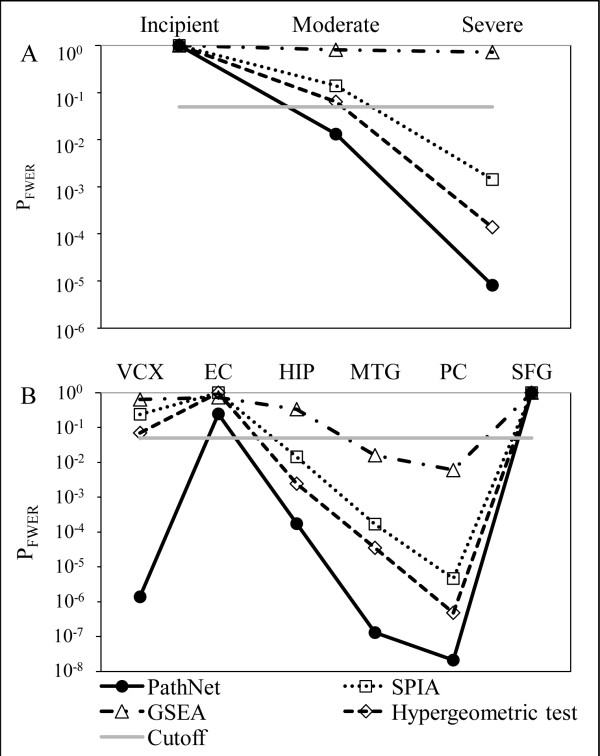
**Enrichment results for the AD pathway.** Enrichment results (p_FWER_: family-wise error rate corrected p-values) for the AD pathway using four different methods in the (**A**) *disease progression dataset* and (**B**) *brain regions dataset* (VCX: primary visual cortex, EC: entorhinal cortex, HIP: hippocampal field CA1, MTG: middle temporal gyrus, PC: posterior cingulate cortex, and SFG: superior frontal gyrus). Only PathNet could identify the AD pathway as significant in the moderate stage of the disease (**A**), and the VCX region (**B**).

Our primary aim was to determine if these methods could identify whether the AD pathway (KEGG ID: 5010) is significantly enriched in AD patients vs. control patients. Figure 3 shows the degree of enrichment of the AD pathway for each of the comparisons, as measured by p_FWER_. Figure 3A shows that using the *disease progression dataset,* none of the methods could identify significant enrichment in the AD pathway during the early (incipient) stages of the disease. As the disease progresses, the significance of the enrichment increased in all four methods. During the late (severe) stages of the disease, three of the four methods could identify significant enrichment in the AD pathway. Notably, at moderate stages of the disease, only PathNet was able to determine that the AD pathway was significantly enriched in AD patients.

In the *brain regions dataset*, all of the methods could identify significant enrichment of the AD pathway in the middle temporal gyrus region and posterior cingulate cortex regions, however, none identified AD enrichment in the entorhinal cortex or superior frontal gyrus regions (Figure 3B). One plausible reason is that the entorhinal cortex samples were from patients with incipient disease. Interestingly, only PathNet could identify significant enrichment of the AD pathway in the primary visual cortex. There is strong evidence in the literature that the primary visual cortex region is indeed affected by AD [22,23]; hence, this is likely not a false positive finding. In each of the comparisons, PathNet consistently yielded the lowest p-value (p_FWER_) for the AD pathway*.*

To test the sensitivity of PathNet with respect to the other three pathway analysis methods, we compared the enrichment levels of seven pathways that have been frequently associated with AD in the literature. Table 1 shows the results from the three stages of the disease using the *disease progression dataset*, with samples taken from the hippocampus region of the brain, and the results in the *brain regions dataset*, with samples from the hippocampal field CA1. PathNet correctly identified most of these pathways as significantly enriched while the other three methods failed to do so. The complete set of results is provided in Additional file 3, which corroborates the favorable performance of PathNet.

**Table 1:** Enrichment of pathways associated with AD

**Pathway (KEGG ID)**		**PathNet**				**SPIA**				**GSEA**				**Hypergeometric test**			
		**Inc**	**Mod**	**Sev**	**HIP**	**Inc**	**Mod**	**Sev**	**HIP**	**Inc**	**Mod**	**Sev**	**HIP**	**Inc**	**Mod**	**Sev**	**HIP**
AD pathway	(5010)	1.00	*0.01	*0.00	*0.00	1.00	0.14	*0.00	*0.01	1.00	0.81	0.72	0.34	1.00	0.06	*0.00	*0.00
Focal adhesion	(4510)	1.00	*0.00	*0.00	*0.00	1.00	1.00	1.00	0.28	1.00	1.00	1.00	1.00	1.00	1.00	1.00	1.00
Long-term pote…	(4720)	1.00	*0.00	*0.01	*0.04	1.00	1.00	1.00	1.00	1.00	1.00	1.00	0.37	1.00	1.00	1.00	1.00
Regulation of a…	(4810)	*0.00	*0.00	*0.00	*0.00	1.00	1.00	1.00	1.00	1.00	1.00	1.00	1.00	1.00	1.00	1.00	1.00
Phosphatidylino…	(4070)	1.00	0.11	*0.00	0.52					1.00	1.00	0.54	1.00	1.00	1.00	0.21	1.00
Wnt signaling	(4310)	*0.00	1.00	*0.00	*0.03	1.00	1.00	0.92	1.00	1.00	1.00	1.00	0.91	1.00	1.00	1.00	1.00
Adherens junct…	(4520)	1.00	*0.00	0.13	*0.00					0.95	0.31	0.96	0.38	1.00	1.00	0.48	*0.01
Ubiquitin media…	(4120)	1.00	1.00	1.00	0.13					1.00	1.00	1.00	0.92	1.00	1.00	1.00	0.17

Enrichment (p_FWER_: family-wise error rate corrected p-values) of pathways associated with Alzheimer’s disease (AD) using four different pathway analysis methods (i.e., PathNet: the present study, *SPIA*: signaling pathway impact analysis, *GSEA*: gene set enrichment analysis, and the hypergeometric test), from the *disease progression dataset* (Inc: incipient, Mod: moderate, and Sev: severe) and from the *brain regions dataset* (*HIP*: hippocampal field CA1 region). The complete set of data is included in Additional files 3, Additional files 4, and Additional files 5. The statistically significant p_fwer_-values (p_fwer_ < 0.05) for each pathway and method are indicated by an asterisk (*). PathNet was able to identify these pathways as significant more often than each of the other three methods. SPIA was not applicable (represented by missing enrichment scores) when certain topological characteristics of the pathway was not met.

To test the specificity of PathNet, we investigated the biological relevance of pathways co-enriched with the AD pathway. Table 2**s**hows that in six out of the nine comparisons where the AD pathway was enriched, we analyzed pathways co-enriched with the AD pathway. Eight pathways were co-enriched with the AD pathway in five or more of the six cases. Of these eight pathways, six were related either to AD (regulation of actin cytoskeleton; adherens junction; focal adhesion; and long-term potentiation) or to other neurological diseases (Parkinson’s disease and Huntington’s disease). Both the Parkinson’s disease pathway and the Huntington’s disease pathway show significant overlap with the AD pathway, which explains why they were frequently co-enriched. There is evidence in the literature to support the association of each of these co-enriched pathways with AD. This qualitatively implies that most of the significantly enriched pathways identified by PathNet are unlikely to be biological false positives.

**Table 2:** Pathways co-enriched with the AD pathway

**Frequency**	**Pathway name**	**(KEGG ID)**	**References**
6	*Bacterial invasion…	(5100)	[24]
6	*Regulation of actin…	(4810)	[25]
5	*Adherens junction	(4520)	[26]
5	*Focal adhesion	(4510)	[27-29]
5	*Huntington's disease	(5016)	[30]
5	*Long-term potenti…	(4720)	[31,32]
5	*Parkinson's disease	(5012)	[30,33]
5	*Pathogenic *Escher*…	(5130)	NA
4	Endocytosis	(4144)	[34]
4	Melanoma	(5218)	[35-37]
4	Pathways in cancer	(5200)	[35-37]
4	Shigellosis	(5131)	NA
3	ECM-receptor	(4512)	[38]
3	Endometrial cancer	(5213)	[35-37]
3	ErbB signaling	(4012)	[39]
3	Fc gamma R-	(4666)	[31]
3	Glioma	(5214)	[40]
3	MAPK signaling	(4010)	[41]
3	Phosphatidylinosit…	(4070)	[42,43]
3	Progesterone-med…	(4914)	NA
3	Proteasome	(3050)	[44,45]

List of pathways co-enriched with the Alzheimer’s disease (AD) pathway in the six out of nine comparisons (moderate and severe samples in the disease progression dataset; and primary visual cortex, hippocampal field CA1, middle temporal gyrus, and posterior cingulate cortex regions in the brain regions dataset) where the AD pathway is enriched. The ‘Frequency’ column shows the number of times the pathway was co-enriched. Pathways that are frequently co-enriched (frequency ≥ 5) are indicated by an asterisk (*). The ‘References’ column provides support for the association of each of these co-enriched pathways with AD. NA: not available.

**Figure 4:**
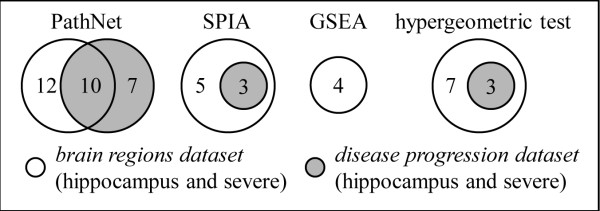
**Overlap of pathways.** Number of enriched pathways that overlap between two comparable datasets (hippocampal field CA1 region for patients with severe AD), using four different methods (i.e., PathNet: the present study, SPIA: signaling pathway impact analysis, GSEA: gene set enrichment analysis, and the hypergeometric test). As an example, PathNet identified 22 and 17 statistically significant pathways from the *brain regions dataset* and the *disease progression dataset*, respectively. Ten of these pathways overlapped. The statistical significances of the overlaps from each of the four methods were tested using the hypergeometric test; p-values were as follows: p_PathNet_ = 2.0 × 10^-5^, p_SPIA_ = 1.5 × 10^-4^, p_GSEA_ = 1.0, p_hypergeometric_ = 3.3 × 10^-4^.

The samples from the *disease progression dataset* were collected from the hippocampal field CA1 region. Similarly, the *brain regions dataset* provides results of samples for patients with severe disease with samples also collected from the hippocampal field CA1 region. Therefore, the data from these two samples, collected in the hippocampus for severe AD patients, should be comparable and the overlap of their significantly enriched pathways can be considered as a measure of the quality of the pathway analysis methods. Figure 4 shows the number of significantly enriched pathways from each dataset and their overlaps. We used the hypergeometric test to compute the significance of the overlap, where the results suggest that PathNet yielded the highest level of significance in overlap when compared to the other methods.

In summary, we compared the results obtained when using PathNet for pathway analysis vs. the results obtained with three existing widely used methods. We found that PathNet was able to: *1)* identify the AD pathway as significant in cases where the existing methods failed; *2)* detect significantly enriched pathways that are known to be biologically relevant to AD; and *3)* detect a higher level of significance in overlap of the enriched pathways in two independent datasets that are expected to be comparable.

### Estimation of false positive rates

We verified that PathNet’s identification of pathways was driven by the differential gene expression data - and not only from the inherent connectivity of the pathways themselves - by testing the performance of PathNet on randomized input data. In the severe stage of the *disease progression data,* we randomly shuffled the gene names 1,000 times and estimated the p_FWER_ values for 130 pathways from PathNet. The randomization of gene names ensures that the direct evidences and number of differentially expressed genes in the shuffled data is the same as in the original data. The distribution of p_FWER_ values given in Additional file 6 show that false positive rates from PathNet were low because 95% of the p_FWER_ values were equal to 1. The false positive rate of PathNet at a p_FWER_ cutoff of 0.05 (used in our analysis) was 0.02. We further investigated if the difference in pathway topology contributes to variations of false positive rates among pathways. We calculated false positive rates for each pathway from 1,000 random shuffles and plotted the distribution of false positive rates for 130 pathways (Additional file 7). The maximum false positive rate was 0.07, implying that none of the pathways have a significantly high probability of being identified as a false positive. Hence, we cannot consider PathNet’s results to be an artifact of the pathway definitions themselves.

### Contextual association between pathways

In this study, we introduced the concept of a contextual association between pathways, i.e., pathway connections that are influenced by differential gene expression of neighboring genes rather than just the static overlap of genes in pathways (Figure 2). Unlike the case of static overlap, these associations are specific to, and dependent on, the biological conditions of the particular study. These calculations identify pathway pairs where the differentially expressed genes linked to each other in the two pathways are present at a greater frequency than would be expected by chance alone.

We used PathNet to identify pathway associations in each of the two AD datasets described above. Because we are interested in analyzing datasets related to AD, we specifically analyzed pathways that have statistically significant contextual association with the AD pathway. We focused on six comparisons (moderate and severe samples in the *disease progression dataset*; and primary visual cortex, hippocampal field CA1, middle temporal gyrus, and posterior cingulate cortex regions in the *brain regions dataset*), where PathNet identified the AD pathway as statistically enriched. The results from all comparisons are provided in Additional file 8. Among the AD contextually associated pathways, Table 3 lists the most frequently appearing pathways in these six comparisons (selected as occurring at least three times). We identified six pathways from this list that are related to neurological disorders in general and AD in particular: gonadotropin releasing hormone (GnRH) signaling; neurotrophin signaling; long-term potentiation; Huntington’s disease; long-term depression; axon guidance; and ubiquitin-mediated proteolysis. GnRH regulates the release of luteinizing hormone, which is elevated in AD patients. The luteinizing hormone is known to be involved in the formation of beta amyloid (Aβ), which is a pathological hallmark of AD [46,47], and the neurotrophin signaling pathway regulates the signaling of neurons [48]. In AD and other neurodegenerative conditions, neurotrophin receptors (NTRs), such as p7NTR, bind to Aβ and nerve growth factors to promote cell death [49]. However, only two of these six pathways (long-term potentiation and Huntington’s disease) were identified as co-enriched (in at least three out of six cases) in the pathway enrichment analysis (Table 2).

**Table 3:** Contextual association of pathways

**Frequency**	**Pathway name**	**(KEGG ID)**	**Overlap**	**References**
			**(p-value)**	
5	Gap junction	(4540)	0.00	[50,51]
5	GnRH signaling…	(4912)	0.00	[46,47,52]
5	Huntington’s…	(5016)	0.00	[30]
4	Adherens junction	(4520)	0.78	[26]
4	Axon guidance	(4360)	0.01	[31,53]
4	Dorso-ventral	(4320)	0.24	NA
4	Insulin signaling	(4910)	0.03	[31]
4	Long-term depression	(4730)	0.00	[54,55]
4	Long-term potentiation	(4720)	0.00	[31,32]
4	Neurotrophin signal…	(4722)	0.01	[31,48]
4	Oocyte meiosis	(4114)	0.00	NA
4	Pathways in cancer	(5200)	0.71	[35-37]
4	Ubiquitin media…	(4120)	1.00	[56,57]

Pathways that were contextually associated [p < 0.05 calculated from Eq. (6)] with the AD pathway from six comparisons (moderate and severe samples in the disease progression dataset; and primary visual cortex, hippocampal field CA1, middle temporal gyrus, and posterior cingulate cortex regions in the brain regions dataset). For comparison purpose, the fourth column shows the p-value of overlapping genes of a pathway and the AD pathway based on hypergeometric test. The ‘References’ column in the table provides support for the association each of the pathways to AD. NA: not available.

**Figure 5:**
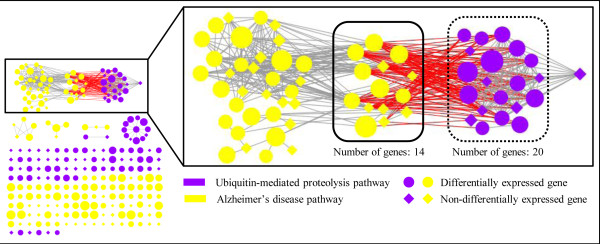
**Connection between Alzheimer’s disease and ubiquitin-mediated proteolysis.** Differential gene expression levels from the posterior cingulate cortex region were overlaid on the Alzheimer’s disease (yellow) and ubiquitin-mediated proteolysis (blue) pathways. The region of the pathways that are directly and indirectly connected to each other is framed and also shown enlarged in the figure. The size of a node (representing a gene) represents the extent of differential gene expression between patients with the disease vs. controls. Although there are no common genes between these two pathways, 112 edges were observed that connected nodes (genes) between the two pathways (highlighted in red) based on inter-pathway connectivity derived from the *pooled pathway*. Several of these edges connect differentially expressed genes in these pathways, analogous to scenario (C) in Figure 2.

If two pathways have significant overlap, i.e., they share a large number of genes, there is an increased chance that they will be associated with each other. However, contextual association is dependent not only on the extent of overlap, but also on the differential expression levels of genes that connect the two pathways. To investigate if the contextual association provided information beyond what could be expected by simply analyzing the shared genes between the corresponding pathway and the AD pathway, we calculated the p-value of the direct overlap of genes in each pathway with the AD pathway, using the hypergeometric test (Table 3). A low p-value indicates that the pathway has a significantly high overlap with the AD pathway, and that the pathways are strongly associated with each other based on previous knowledge encoded in the pathway definitions themselves. Interestingly, in 31% of the cases we observed that pathways with limited overlap had significant contextual association with each other. For example, ubiquitin-mediated proteolysis is one of the pathways that do not share any genes with the AD pathway, and yet we found that, in four out of six comparisons, this pathway was contextually associated with the AD pathway (Table 3, Column 4). We therefore investigated the relationship between the AD and ubiquitin-mediated proteolysis pathways further. Figure 5 shows that there are 112 edges connecting genes between these two pathways, which imply a possible association between them. However, because these edges connect genes from two non-overlapping pathways, we could not have identified this relationship if we had treated the pathways separately, or if we had used methods that relate pathways based solely on overlapping genes. It is well established that deregulation of ubiquitin-mediated proteolysis can lead to the formation of neurofibrillary tangles (NFTs) from hyper-phosphorylated tau protein [31,56,57]. NFTs are one of the pathological hallmarks of AD, and the number of NFTs increases with the progression of the disease [31]. However, this biologically relevant pathway is not statistically enriched from any of the four pathway analysis methods used here (Table 1), suggesting that our contextual association between pathways can distil biological information that could not be obtained from enrichment analysis alone.

In summary, the following observations were made: 1) enrichment analysis using PathNet performed better than the three existing pathway analysis methods in identifying biologically relevant pathways, 2) contextual pathway-pathway analysis can reveal biological insights that may not be obtained from enrichment analysis alone, and 3) the enrichment of pathways associated with AD changes with disease progression.

## Conclusion

In this study, we developed PathNet, a method for pathway analysis based on high-throughput molecular profiling data, using inter- and intra-pathway connectivity information. PathNet calculates both pathway enrichment and contextual associations between pathways. We have shown that PathNet was able to identify the AD pathway and other biologically relevant pathways in multiple scenarios while three other widely used pathway analysis methods (hypergeometric test, GSEA, and SPIA) often failed to do so. PathNet also identified pathways contextually associated with the AD pathway. Literature studies support the biological relevance of the results identified using PathNet.

The existing methods used for pathway enrichment consider each pathway as a separate entity. In contrast, PathNet considers both inter-pathway and intra-pathway connectivity for pathway enrichment. This connectivity information, in the form of a significance-level weighted gene-gene connection, corroborates and strengthens the direct evidence of differential gene expression readily derived from microarray data when a gene’s neighbors on the pathway are also differentially expressed. The method properly accounts for highly connected genes that are part of multiple pathways via comparison with the appropriate probability density function generated from topology-preserving randomized data. The unbiased nature of this method was confirmed by the estimated low false positive rates. However, if no connectivity information is available for a gene, PathNet still includes the microarray-derived evidence for identifying pathway enrichment. This ensures that we do not penalize genes that have no information available regarding their connectivity.

In PathNet, indirect evidence of a gene is calculated based on gene expression levels of its neighbors using Eqs. (1–3). Hence, indirect evidence of the gene cannot be estimated if neighboring gene expression is not measured in the microarray analysis. In such cases, the combined evidence of a gene is replaced with the direct evidence. In the limiting case where none of the genes’ neighbors expression levels are measured, PathNet converges to a standard hypergeometric test.

Currently, there is no gold standard for quantitatively testing and comparing the performance of pathway enrichment methods. As an alternative, we have selected a well-studied disease (i.e., AD), where considerable amount of knowledge already exists about the deregulation of its biological processes and multiple high-quality microarray datasets are available, to examine important aspects of the disease. This allowed us to assess the performance of PathNet based on an in-depth analysis of the biological relevance of the results, directly compare its performance with other existing pathway enrichment methods, and ascertain each method’s ability to retrieve the relevant biological information.

### Availability and requirements

**Software name:** PathNet

**Download site:**http://www.bhsai.org/downloads/pathnet/

**Operating system:** Platform independent

**License:** GPL version 3

**Programming language:** R version 2.14.1 or later

## Abbreviations

Aβ: Beta amyloid; AD: Alzheimer’s disease; EC: Entorhinal cortex; GEO: Gene expression omnibus; GSEA: Gene set enrichment analysis; GnRH: Gonadotropin releasing hormone; GO: Gene Ontology; HIP: Hippocampal field CA1; KEGG: Kyoto encyclopedia of genes and genomes; MTG: Middle temporal gyrus; NFTs: Neurofibrillary tangles; NTRs: Neurotrophin receptors; PC: Posterior cingulate cortex; PPI: Protein-protein interaction; SFG: Superior frontal gyrus; SPIA: Signaling pathway impact analysis; VCX: Primary visual cortex.

## Competing interests

The authors declare that they have no competing interests.

## Authors' contributions

BD, AW, and JR conceived of the algorithm. BD implemented the algorithm, performed the study, and wrote the first draft of the manuscript. All authors contributed to the manuscript writing and approved the final manuscript.

## Supplementary Material

Additional file 1 **KEGG directionality assignments.** This file gives the types of edge directionality used in the KEGG pathway. Click here for file

Additional file 2 **Scatter-plots of direct and indirect evidences.** A figure showing the relationship between direct and indirect evidences for the nine different comparisons used in this work. Click here for file

Additional file 3 **Hypergeometric test and PathNet results.** An Excel spreadsheet of the results of all nine comparisons using the hypergeometric test and PathNet. Click here for file

Additional file 4 **GSEA results.** An Excel spreadsheet of the results of all nine comparisons using GEAS. Click here for file

Additional file 5 **SPIA results.** An Excel spreadsheet of the results of all nine comparisons using SPIA. Click here for file

Additional file 6 **Randomized distributions of p**_**FWER**_**.** Distribution of p_FWER_ from PathNet derived from the null distribution scenario and obtained from data randomization. Click here for file

Additional file 7 **Estimated false positive rate.** Distribution of estimated false positive rates based on an analysis of all pathways. Click here for file

Additional file 8 **Contextual AD pathway association.** An Excel spreadsheet of the pathways identified to have a statistically significant contextual association with the AD pathway. Click here for file
