# Supplementary material for: PathNet: a tool for pathway analysis using topological information
Source: Source Code Biol Med. 2012 Sep 24;7:10. doi: 10.1186/1751-0473-7-10 (PMC3563509; doi:10.1186/1751-0473-7-10)
Supplement: Additional file 1 — KEGG directionality assignments. This file gives the types of edge directionality used in the KEGG pathway. [file 1751-0473-7-10-S1.docx]

Additional file 1: Directionality of the edges in KEGG pathway

| **Interaction type** | **Directionality of the edge** |
| --- | --- |
| Compound | Bidirectional |
| Activation | Unidirectional |
| Inhibition | Unidirectional |
| Expression | Unidirectional |
| Repression | Unidirectional |
| Indirect effect | Unidirectional |
| State change | Bidirectional |
| Binding / association | Bidirectional |
| Dissociation | Bidirectional |
| Missing Interaction | Bidirectional |
| Phosphorylation | Unidirectional |
| Dephosphorylation | Unidirectional |
| Ubiquitination | Unidirectional |
