# Supplementary material for: PathNet: a tool for pathway analysis using topological information
Source: Source Code Biol Med. 2012 Sep 24;7:10. doi: 10.1186/1751-0473-7-10 (PMC3563509; doi:10.1186/1751-0473-7-10)

Additional file 2: Scatter-plots of direct and indirect evidences

We plotted direct and indirect evidences for nine different comparisons used in our analysis (details of the comparisons are provided in Microarray datasets Section). In all of the comparisons, there is no obvious relationship between direct and indirect evidences.


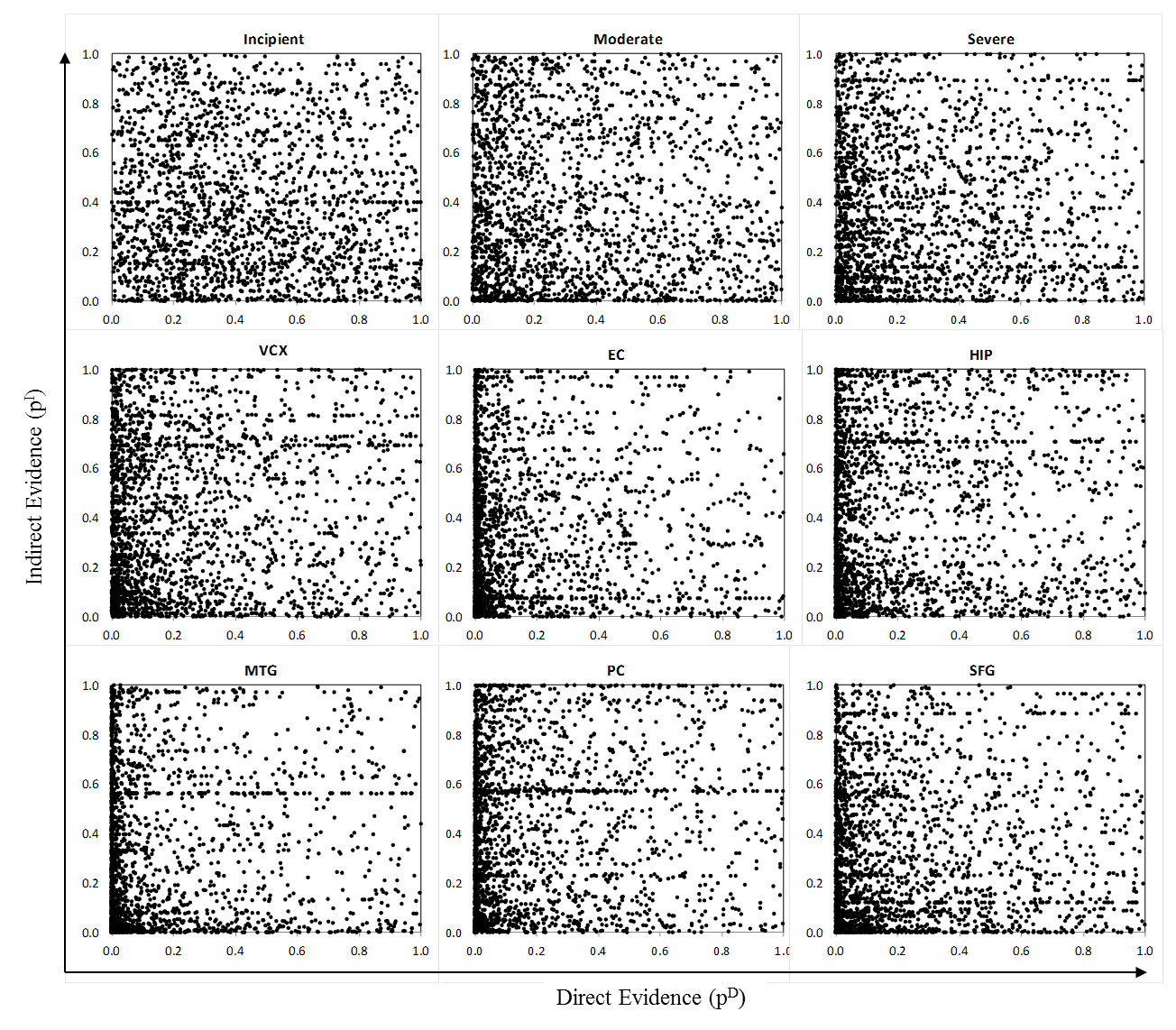

Supplement: Additional file 2 — Scatter-plots of direct and indirect evidences. A figure showing the relationship between direct and indirect evidences for the nine different comparisons used in this work. [file 1751-0473-7-10-S2.docx]
