# Supplementary material for: PathNet: a tool for pathway analysis using topological information
Source: Source Code Biol Med. 2012 Sep 24;7:10. doi: 10.1186/1751-0473-7-10 (PMC3563509; doi:10.1186/1751-0473-7-10)

Additional file 6: Randomized distributions of p_FWER_

Distribution of p_FWER_ from PathNet derived from the null distribution scenario and obtained from data randomization. In the severe stage of the *disease progression dataset*, gene names were randomly shuffled 1,000 times and PathNet was used to estimate the distribution of the p_FWER_ for each of the 130 pathways. Results were pooled to create a distribution from 130,000 p_FWER_ samples. When gene names are randomly shuffled, we do not expect any pathway to become significant. Hence, the number of significant pathways from the randomly shuffled data provides a measure of the false positive rates. The distribution shows that the false positive rates are extremely low. In fact, in 95% of the cases, p_FWER_ was equal to 1. Mean and median values of this distribution are 0.96 and 1, respectively. Only 2.14% samples from this distribution have p_FWER_ less than 0.05, which is the cutoff we used in our analysis.


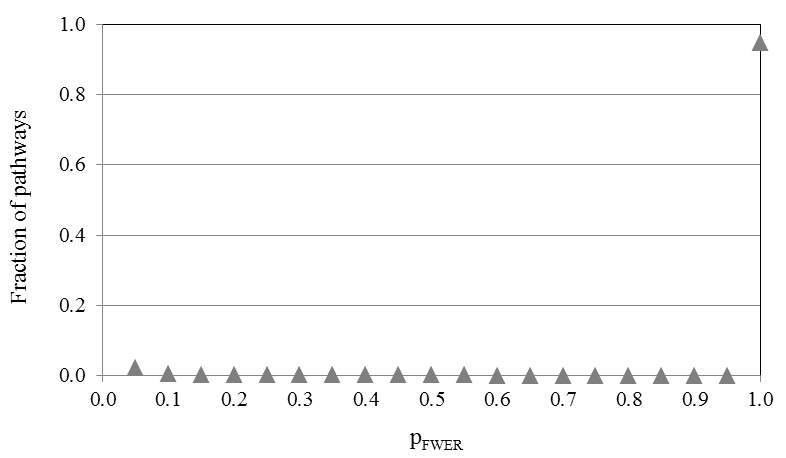

Supplement: Additional file 6 — Randomized distributions of pFWER. Distribution of pFWER from PathNet derived from the null distribution scenario and obtained from data randomization. [file 1751-0473-7-10-S6.docx]
