# Supplementary material for: PathNet: a tool for pathway analysis using topological information
Source: Source Code Biol Med. 2012 Sep 24;7:10. doi: 10.1186/1751-0473-7-10 (PMC3563509; doi:10.1186/1751-0473-7-10)

Additional file 7: Estimated false positive rate

To study the variation of false positive rates among pathways, we estimated the false positive rates for each of the 130 pathways separately. For each of the pathways, we estimated false positive rates by counting the fraction of randomizations which falsely identifies the pathway as significant (i.e., p_FWER_ < 0.05). Minimum and maximum false positive rates were 0 and 0.07, respectively. Hence, none of the pathways has a high probability of becoming falsely identified as significant due to the inherent topology of the pathway. PathNet identified Alzheimer’s disease pathway as significant in the severe stage of the *disease progression dataset*. Estimated false positive rate of this pathway was 0.002 based on randomization, implying that it is highly improbable that this pathway was falsely identified as significant.


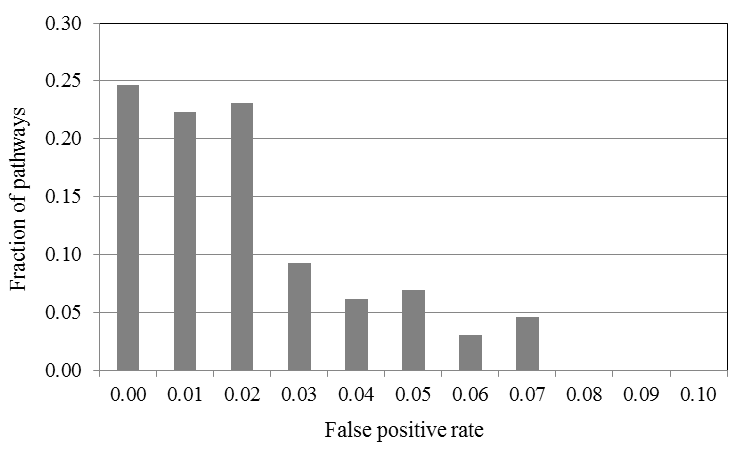

Supplement: Additional file 7 — Estimated false positive rate. Distribution of estimated false positive rates based on an analysis of all pathways. [file 1751-0473-7-10-S7.docx]
